# Endoscopic Third Ventriculostomy in a 12-Year-Old With Recurrent Failure of Ventriculoperitoneal Shunts

**DOI:** 10.7759/cureus.38270

**Published:** 2023-04-28

**Authors:** Monica Dhiman, Fahim Soukhak, Jonathan Eskenazi

**Affiliations:** 1 Family Medicine, HCA (Hospital Corporation of America) Medical City, Arlington, USA; 2 Neurology, Ross University School of Medicine, Miramar, USA; 3 Neurology, Cedars-Sinai Medical Center, Los Angeles, USA

**Keywords:** vp shunt, cerebrospinal fluid (csf), shunt malfunction, congenital hydrocephalus, endoscopic ventriculostomy, vp shunt complication

## Abstract

With a success rate of about 80%, ventriculoperitoneal (VP) shunts are widely used for the treatment of hydrocephalus. Whether congenital or acquired, hydrocephalus is not a single disease entity. It can be caused by abnormal cerebrospinal fluid (CSF) reabsorption, obstruction along the ventricular pathways, or, very rarely, increased production of CSF itself. This case presents a patient with a history of congenital hydrocephalus with multiple failed VP shunts. Through various clinical examinations and diagnostic measures, an endoscopic third ventriculostomy was eventually performed. This case highlights the rare complications, yet a large possibility, that can lead to failure of VP shunts in more than one way and when it is appropriate for shunt reversal versus removal.

## Introduction

Congenital hydrocephalus has had a recent rise in low to mid-income regions, with an incidence of 123 per 100,000 live births. Given the high crude birth rate and post-infectious etiology, the increased burden that Southeast Asia and Latin America has is incomparable to the Western world [1,2]. Hydrocephalus itself can be defined by either absorption or production malfunction. Congenital hydrocephalus, caused more so by excessive cerebrospinal fluid (CSF) production, stems from either a brain malformation or a defect in the ventricular system. To overcome these malfunctions, medicine has procured the use of ventriculoperitoneal (VP) shunts. VP shunts consist of a ventricular catheter with the distal end placed in the peritoneal cavity [3]. This functions as a drain to get rid of the excess CSF. If left untreated, pediatric patients can be left with chronic conditions such as headaches, learning disabilities, visual disturbances, and severe mental disabilities [3]. Although VP shunts are known to have an 80% success rate, these can often malfunction, leading to an increased accumulation of CSF. This can lead to greater chances of cerebral edema and herniation due to increased intracranial pressure [3]. 

There are numerous forms of shunt malfunctions and complications, something our patient in this case experienced in more than one way. Complications include malpositioning, infection, hemorrhage, obstruction, shunt disconnection, abdominal CSF collection (in the form of ascites), peritonitis, and countless more [3]. These malfunctions present with acute symptoms such as nausea, vomiting, headache, lethargy, diplopia, fever, neck rigidity, and tense fontanelle [3]. If such symptoms present, a proper assessment of shunt function has to be done through the shunt and CSF tapping. The opening pressure can be measured via a manometer and the CSF sample can be sent for cell count, glucose, protein, Gram stain, and cultures for assessment of any infectious etiology [3]. Here, we have a rather complicated case that presented with the aforementioned symptoms with multiple VP shunt failures. 

## Case presentation

A 12-year-old female with a medical history of congenital hydrocephalus diagnosed at two months of age and surgical history of four malfunctioning VP shunts was admitted to the hospital after presenting with a severe headache. The patient also endorsed multiple recent episodes of non-bilious/non-bloody projectile vomiting and headache but denied photophobia, blurry vision, fevers, and coughs. The physical exam showed mild tachycardia; however, fundal, neurological, and musculoskeletal exams were all within normal limits. No bilateral papilledema was appreciated. The patient was started on IV fluids (3% sodium chloride (NaCl) and dextrose normal saline (DNS)) for dehydration, ondansetron for anti-emetic effects, and acetazolamide to reduce intracranial pressure. Even after the administration of treatments, the patient's condition was not improving and still presented with persistent vomiting and headache. A neurosurgical investigation was consulted and the patient was also given an injection of ceftriaxone for empirical measures. As per the neurosurgical consult, CSF was drained from the left-sided VP shunt and sent for investigation. In the next few days, the patient continued to remain afebrile and vitally stable; however, at the same time, she started to present with abdominal pain and distention. This led us to suspect a possible build-up of CSF in the peritoneum. A bedside ultrasound was conducted which confirmed the build-up of fluid in the abdomen/peritoneal region. Tapped fluid studies showed the presence of CSF, indicating another failure of the patient’s VP shunt. The patient was then started on Lasix (furosemide), shunt pumping was halted, and the patient was prepared for pre-operative measures. Within two days, the patient started having fever spikes between 102F to 104F. She was started on cefixime, glycerol, and paracetamol for supportive measures. 

**Figure 1:**
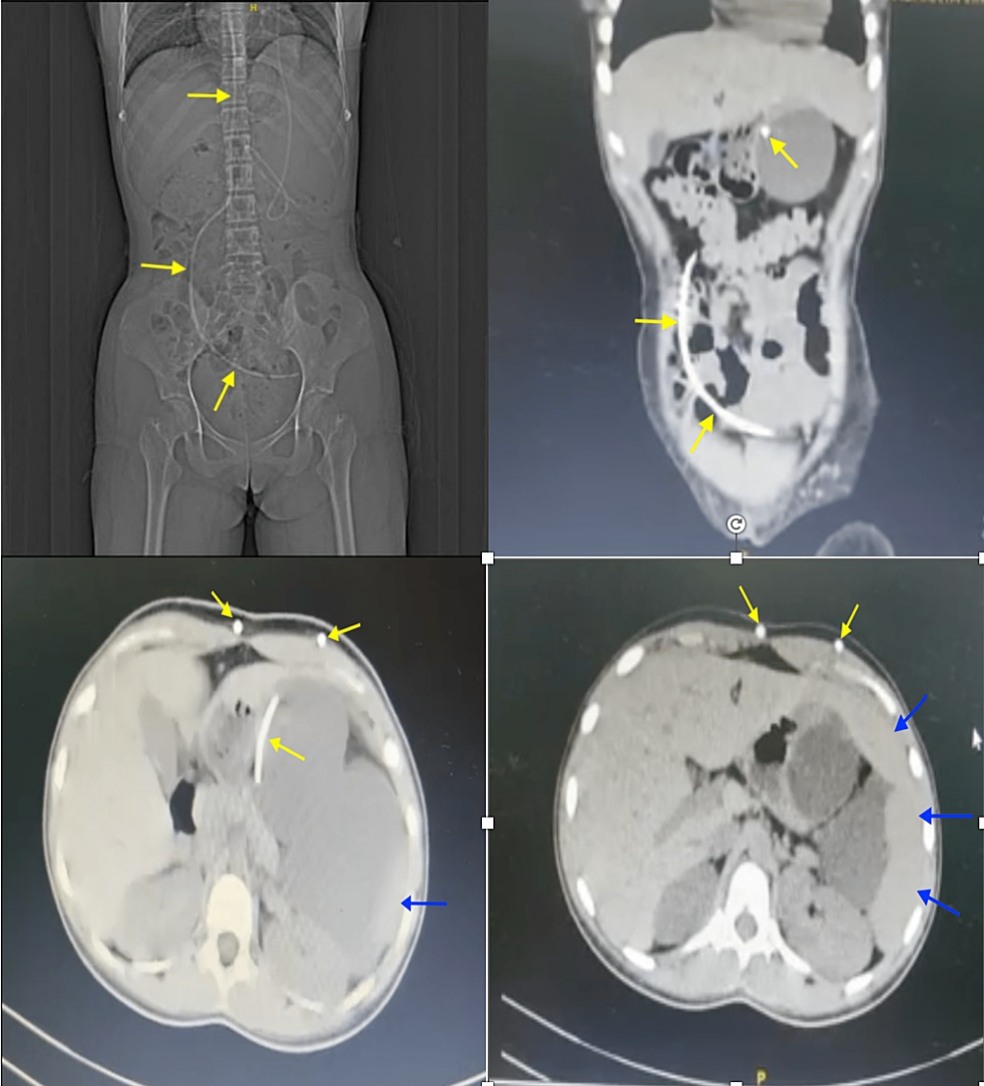
Long catheter of VP shunt in abdominal cavity (yellow arrows), Fluid accumulation on the left hypochondriac region (blue arrows).

**Figure 2:**
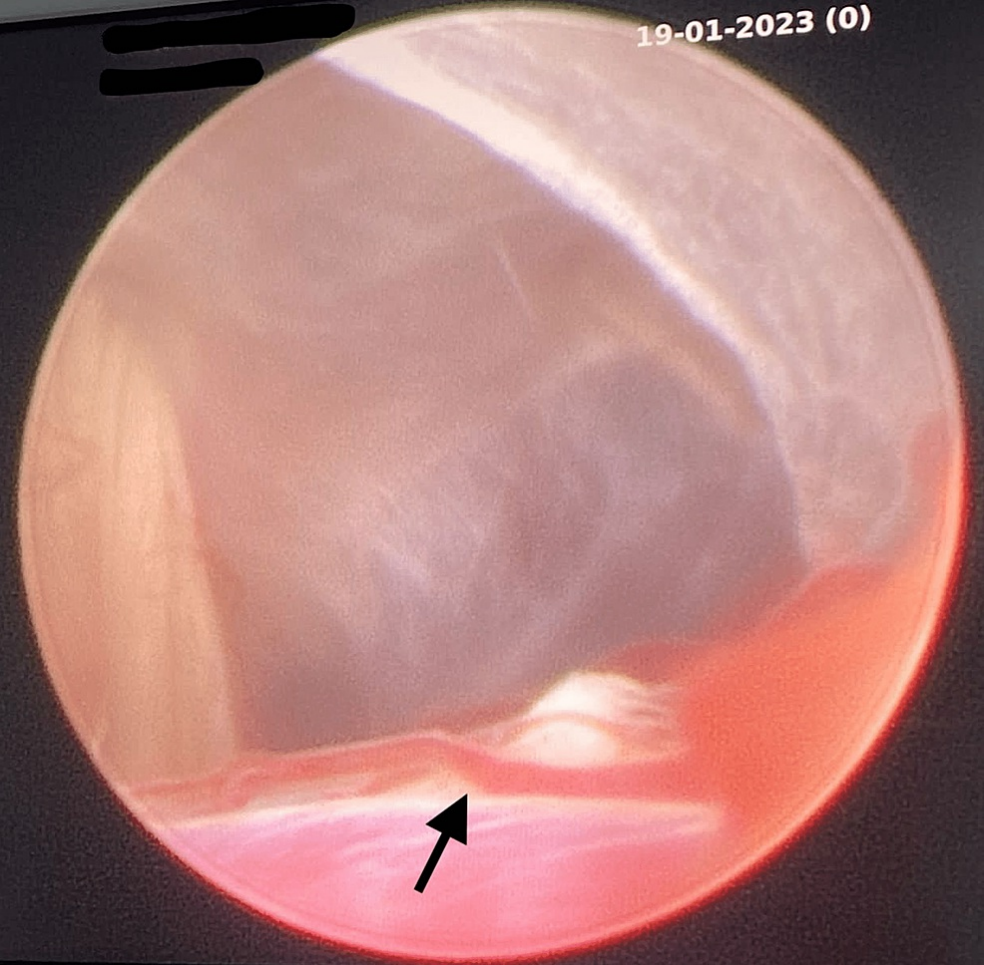
Orifice created in the third ventricle during the procedure; the basilar artery can be visualized as well (black arrow).

CT scan was conducted, which revealed the VP shunt tip in the abdominal end and the build-up of fluid in the left hypochondriac region (Figure 1). Per the neurosurgery consult, shunt revision had to be conducted as soon as possible. The patient was then prepped for pre-operative evaluation and taken in for the surgical procedure where an endoscopic third ventriculostomy (ETV) was conducted (Figure 2). Injection of ceftriaxone and sulbactam was administered to the patient while she underwent the procedure.

The patient tolerated the procedure well and was shifted to the pediatrics intensive care unit for close observation. After supportive management and close observation, the patient had a stable post-operative recovery and was discharged within two weeks with further management and follow-up instructions given in the next few weeks.

## Discussion

In this case, our patient comes from a low-income rural Indian background and faced recurrent VP shunt failures, five to be exact, with multiple shunt reconstructions. When the patient presented to us, she showed signs of CSF ascites, peritonitis, and several acute symptoms; all pointing towards one more shunt failure. Ascites resulting from CSF accumulation is a rare complication in itself, but in this case, not surprising. The peritoneum is burdened with an excess of CSF and is, therefore, unable to absorb all of the fluid [4]. Normally, the rate of fluid absorption correlates with the pressure, however, such as in this case, an increase in the rate of CSF production will also increase the intracranial pressure. This causes the shunt drain to have increased CSF flow into the peritoneal cavity, leading to ascites [5]. Having presented with such signs and after a consult with neurosurgery, the next best step for this patient’s hydrocephalus was concluded to be an ETV, a minimally invasive procedure that bypasses the blockage, created an opening, and restores the proper flow of cerebrospinal fluid [6]. Although this procedure is often the next step once VP shunts have failed to aid the patient, it is still quite a risky procedure. This is due to the high chance of injury to the vascular system or in this patient’s case, the basilar artery, as it is so close to the penetration site [7]. During the procedure, even the slightest manipulation would elevate the patient’s heart rate, indicating the high fragility of the vascular system and the opening created in the third ventricle. Although the procedure itself may seem daunting, the postoperative period is the most crucial in such patients. The patient has to often be monitored for weeks to months to ensure a decrease in ventricular size, an indication of a positive clinical outcome [7]. In the majority of cases, as is the trajectory of ours, the patient often has a satisfactory outcome with few postoperative complications. 

## Conclusions

Even with limited resources, this patient and her family have lived and adjusted to congenital hydrocephalus since birth, and facing various forms of recurrent shunt failure has not made it simple. Her family members had been made aware of the signs and symptoms of shunt failure, as it has occurred one too many times. However, with the help of advanced minimally invasive procedures such as ETV, such patients can live a long and complication-free life. It is also crucial to educate such patients and their family members about the signs and symptoms of infection, increased intracranial pressure, and complications that require immediate medical attention.
